# SARS-CoV-2 Infects Red Blood Cell Progenitors and Dysregulates Hemoglobin and Iron Metabolism

**DOI:** 10.1007/s12015-021-10322-8

**Published:** 2022-02-18

**Authors:** Romy Kronstein-Wiedemann, Marlena Stadtmüller, Sofia Traikov, Mandy Georgi, Madeleine Teichert, Hesham Yosef, Jan Wallenborn, Andreas Karl, Karin Schütze, Michael Wagner, Ali El-Armouche, Torsten Tonn

**Affiliations:** 1 Department of Transfusion Medicine, Medical Faculty Carl Gustav Carus, Technische Universität Dresden, Fetscherstraße. 74, 01307 Dresden, Germany; 2 Institute of Medical Microbiology and Virology, Medical Faculty Carl Gustav Carus, Technische Universität Dresden, Fetscherstraße. 74, 01307 Dresden, Germany; 3 Max-Planck-Institute of Molecular Cell Biology and Genetic, Pfotenhauerstr. 108, 01307 Dresden, Germany; 4 Clinic of Anaesthesiology and Intensive Care Medicine, HELIOS Clinic, Gartenstraße 6, 08280 Aue, Germany; 5 CellTool GmbH, Lindemannstraße 13, 82327 Tutzing, Germany; 6 German Red Cross Blood Donation Service North-East, Institute for Transfusion Medicine, Röntgenstraße 2a, 08529 Plauen, Germany; 7 Clinic for Internal Medicine and Cardiology, Heart Center Dresden, Technische Universität Dresden, Fetscherstraße. 76, 01307 Dresden, Germany; 8 Institute of Clinical Pharmacology, Medical Faculty Carl Gustav Carus, Technische Universität Dresden, Fetscherstraße. 74, 01307 Dresden, Germany; 9 German Red Cross Blood Donation Service North-East, Institute for Transfusion Medicine, Blasewitzer Straße. 68/70, 01307 Dresden, Germany

**Keywords:** COVID-19, Erythroid precursors, Hemoglobin biosynthesis, Hemoglobin degradation, Iron metabolism, Mass spectrometry, Raman Trapping Microscopy

## Abstract

**Background:**

SARS-CoV-2 infection causes acute respiratory distress, which may progress to multiorgan failure and death. Severe COVID-19 disease is accompanied by reduced erythrocyte turnover, low hemoglobin levels along with increased total bilirubin and ferritin serum concentrations. Moreover, expansion of erythroid progenitors in peripheral blood together with hypoxia, anemia, and coagulopathies highly correlates with severity and mortality. We demonstrate that SARS-CoV-2 directly infects erythroid precursor cells, impairs hemoglobin homeostasis and aggravates COVID-19 disease.

**Methods:**

Erythroid precursor cells derived from peripheral CD34+ blood stem cells of healthy donors were infected in vitro with SARS-CoV-2 alpha variant and differentiated into red blood cells (RBCs). Hemoglobin and iron metabolism in hospitalized COVID-19 patients and controls were analyzed in plasma-depleted whole blood samples. Raman trapping spectroscopy rapidly identified diseased cells.

**Results:**

RBC precursors express ACE2 receptor and CD147 at day 5 of differentiation, which makes them susceptible to SARS-CoV-2 infection. qPCR analysis of differentiated RBCs revealed increased HAMP mRNA expression levels, encoding for hepcidin, which inhibits iron uptake. COVID-19 patients showed impaired hemoglobin biosynthesis, enhanced formation of zinc-protoporphyrine IX, heme-CO_2_, and CO-hemoglobin as well as degradation of Fe-heme. Moreover, significant iron dysmetablolism with high serum ferritin and low serum iron and transferrin levels occurred, explaining disturbances of oxygen-binding capacity in severely ill COVID-19 patients.

**Conclusions:**

Our data identify RBC precursors as a direct target of SARS-CoV-2 and suggest that SARS-CoV-2 induced dysregulation in hemoglobin- and iron-metabolism contributes to the severe systemic course of COVID-19. This opens the door for new diagnostic and therapeutic strategies.

**Graphical Abstract:**

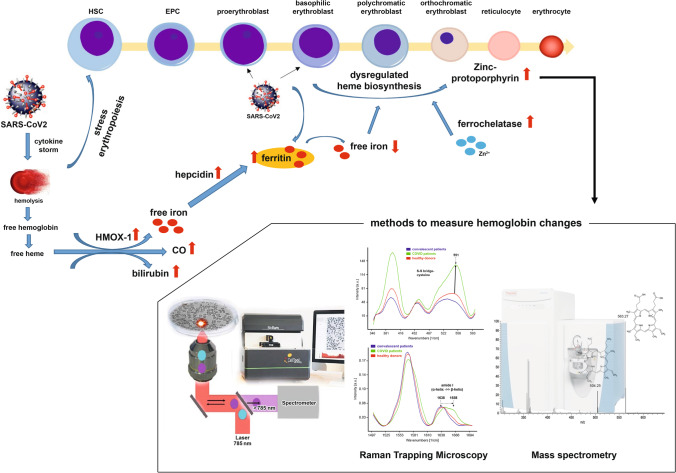

## Introduction

Severe acute respiratory syndrome coronavirus 2 (SARS-CoV-2) is the cause of the worldwide COVID-19 pandemic. While COVID-19 has a mild course in most of the infected, it progresses to severe respiratory failure in a significant number of cases. COVID-19 is increasingly recognized to be a systematic disease where reduced oxygen transport capacity of the blood may play a key role [1]. Severe cases are associated with reduced erythrocyte turnover and low hemoglobin levels along with increased total bilirubin and ferritin concentrations. Furthermore, a significant alteration in the size of the spleen from deceased COVID-19 patients was reported, which is a normal physiological response to anemia [2]. Additionally, reduced numbers of T- and B-lymphocytes as well as natural killer cells in the peripheral blood of COVID-19 patients have been reported, especially in patients with severe disease [3].

The functional and most important role of red blood cells (RBCs) is the transportation of oxygen from the lungs to the tissues providing each individual cell with the required amount of oxygen [4]. Therefore, anemia may play a role in the severity of tissue hypoxia in COVID-19 patients [5]. As such, hemoglobin alteration may compromise oxygen carrying capacity of RBCs in COVID-19 patients resulting in hypoxia.

RBC precursors are characterized by extreme iron requirements to sustain hemoglobin synthesis. This starts already at the stage of basophilic erythroblast, which expresses molecules dedicated to the transport, utilization, and storage of iron [6]. The requirement for iron is mainly satisfied by macrophages, which recycle senescent erythrocytes, as well as by iron uptake with food and the hepatic iron stores. Iron uptake in erythrocytes depends on transferrin, a glycoprotein with two high-affinity sites for Fe (III). Furthermore, transferrin prevents the formation of toxic radicals and restricts iron access for invading pathogens [6].

Based on preliminary computational and genetic sequencing researches, hypotheses about the pathophysiological mechanisms of RBC development include the interaction of SARS-CoV-2 proteins with hemoglobin molecules, leading to hemoglobin dysfunction, as well as tissue iron overload and disrupted heme metabolism [7–9].

Iron metabolism may be involved in the pathophysiology of COVID-19 also via a second potential pathway. It was suggested that the innate immune system may aim to decrease the bioavailability of iron in order to prevent an expanding viral load in the acute phase of the infection. This leads to the activation of hepcidin, sequestration of iron within cells, increased levels of ferritin and decreased hemoglobin, culminating in hypoxia [10]. Together, hemoglobinopathy and iron dysmetabolism may seriously compromise the capacity of erythrocytes to perform O_2_ transport resulting in hypoxia and in addition induce hyperferritinemia-related tissue alterations [7].

ACE2, CD147 and CD26 were identified as the cellular entry receptors for SARS-CoV-2 [11–13]. ACE2 is highly expressed in epithelial cells of the respiratory tract. However, its expression has also been demonstrated in cells from other tissues such as intestinal epithelial cells, hepatocytes, neurons, and bone marrow-derived VSELs and HSCs [14–17]. More recently, an expansion of erythroid progenitor cells in peripheral blood has been reported and together with hypoxia, anemia, and coagulopathies it highly correlates with disease severity and mortality [18]. Erythroid precursors (CD71^+^ erythroid cells, CECs) are also involved in immune regulation. They suppress proinflammatory responses of myeloid cells and T cell proliferation. Moreover, CECs produce reactive oxygen species (ROS) to decrease T cell proliferation, and the production of ROS makes T cells more permissive for virus infection. CECs also secrete cytokines, including transforming growth factor β (TGF-β), which promotes T cell differentiation into regulatory T-cells [19]. Intriguingly, there is first evidence that erythroid progenitors may be directly infected by SARS-CoV-2 [20, 21]. However, the impact of SARS-CoV-2 infection on erythropoiesis has not been well investigated to date. Here, we provide the first experimental evidence of disruption of hemoglobin synthesis and iron dysmetabolism by direct SARS-CoV-2 infection of erythroid progenitors.

## Material and Methods

### Materials

Recombinant human SCF, IL-3 and EPO were from Peprotech (Hamburg, Germany). Insulin solution, holo-transferrin, heparin, hydrocortisone, bovine albumin, crystal violet, and DEAE-Dextran were from Sigma/Aldrich (Schnelldorf, Germany). MgCl_2_, CaCl_2_, NaHCO_3_, and formaldehyde were from Carl Roth (Karlsruhe, Germany).

### Blood Collection and Sampling

3 mL whole blood from healthy donors, intensive-care COVID-19 patients and from patients who had recovered (convalescent donors) were collected in S-Monovettes EDTA K3 (Sarstedt, Nürnbrecht, Germany) by venipuncture using a sterile disposable Safety-Multifly®- Needle 21G (Sarstedt). Determination of red blood cells, hemoglobin content and hematocrit were performed on a Sysmex XN 1000 (Sysmex Deutschland GmbH, Norderstedt, Germany). Measurement of pH value and blood gas analyzing were carried out with an ABL800 Flex (Radiometer Medical ApS, Brønshø, Denmark).

Plasma was collected for measurement of iron metabolism parameters (performed in the Institute of Clinical Chemistry and Laboratory Medicine, Dresden).

### Raman Trapping Microscopy

All Raman measurements were performed using an inverted microscopic Raman system (BioRam®, CellTool GmbH, Tutzing, Germany). The samples were excited using a laser diode at 785 nm wavelength and 60 mw power (TOPTICA Photonics AG, Graefelfing, Germany). Scattered photons were collected using a 60x water immersion objective (1.1 NA, 0.2 WD) (Olympus, Hamburg, Germany) with correction collar set to 0.17 mm. Detection of Raman scattered photons was achieved using diffraction grating and a CCD detector (Andor, Belfast, UK). For measurements, 100 μL of one whole blood sample were applied into a μ-Slide with borosilicate glass bottom (Ibidi GmbH, Martinsried, Germany). For each sample, 100 RBCs were measured. Cells were exposed to the laser for overall 30 s divided on 3 accumulations (3 × 10 s).

### Statistical Analysis of the Raman Data

Spectral data processing and statistical analysis were preformed using the CT-RamSES software (CellTool GmbH, Tutzing, Germany). Raman spectra were cropped to the spectral range of 350–1800 cm^−1^ that contains most of the biologically relevant spectral bands. The baseline was then corrected by an asymmetric least square fit, cosmic spikes were removed, and the spectra were smoothened with a median filter. Finally, the spectra were interpolated to continuous wave numbers and normalized using unit-vector-normalization. Principal component analysis (PCA) score plots were used to visualize differences and similarities among samples while the PCA loadings illuminate the Raman spectral differences used to compare the analyzed samples.

### Isolation, Differentiation of Hematopoietic Stem Cells and Infection of CECs

CD34^+^ cells from peripheral blood were isolated by centrifugation over Biocoll and subsequently magnetic microbead selection of CD34+ mononuclear cells (MNC’s) by the use of LS-MACS columns (Miltenyi Biotec, Bergisch Gladbach, Gemany; 94% ± 3% purity). The cells were cultured as described previously [22]. 1 × 10^4^/mL CD34+ cells were cultured in IMDM (Life technologies, Darmstadt, Germany), supplemented with 1% L-glutamine (Biozym, Hessisch Oldendorf, Germany), 100 units/mL penicillin and 100 μg/mL streptomycin (Biozym), 330 μg/mL human holo-transferrin, 10 μg/mL recombinant human insulin, 2 IU/mL heparin Choay, and 5% pooled AB serum (German Red Cross Blood Donation Service North-East, Dresden, Germany), in the presence of 10^−6^ M hydrocortisone, 100 ng/mL SCF, 5 ng/mL IL-3 and 3 IU/mL EPO. On day 5, cells were infected with a multiplicity of infection (MOI) of 5 by adding SARS-CoV-2 virus hCoV-19/Germany/SN-RKI-I-038776/2021 (B.1.1.7/Alpha) to the medium of one cell culture flask per donor. On day 7, cells were harvested for analysis.

Virus titers were determined using plaque assay. Briefly, confluent Vero E6 cells were washed with PBS and subsequently infected with 10-fold serial dilutions of samples in PBS (supplemented with 0.3% bovine albumin, 1 mM MgCl_2_ and 1 mM CaCl_2_). After 1 h of infection at 37 °C and 5% CO_2_ with occasional shaking, the inoculum was removed. The cells were washed with PBS, overlaid with semi-viscous avicel overlay medium (double-strength DMEM (ThermoFisher Scientific, Dreieich, Germany), 0.75% avicel RC-581 in H_2_O (DuPont, Wilmington, USA), 10% FCS, 0.01% DEAE-dextran and 0.05% NaHCO_3_) and incubated at 37 °C and 5% CO_2_ for 3 days. Plaques were visualized by staining cells with 0.1% crystal violet in 30% formaldehyde for 30 min. Virus titration results are given as plaque forming units per mL (PFU/mL).

### RNA Isolation and Quantitative Reverse Transcription Polymerase Chain Reaction (qRT-PCR)

Total RNA was extracted from 1 mL plasma-depleted whole blood or 2 × 10^7^ cultured CECs using the pax gene blood miRNA kit (PreAnalytiX, QIAGEN, Hilden). Reverse transcription of mRNA was performed using the revert aid H minus first strand cDNA synthesis kit according to the manufacturer’s directions (Fisher Scientific, Schwerte, Germany). Realtime PCR was performed with TaqMan universal PCR master mix according to manufacturer’s instruction using a TaqMan-assay for the hepcidin antimicrobial peptide sequence (HAMP, Hs00221783_m1). GAPDH was used as the endogenous control using the primers forward-5′-AGG GCT GCT TTT AAC TCT GGT AA-3′, reverse-5′- CAT GGG TGG AAT CAT ATT GGA AC-3′ and probe FAM-TGT TGC CAT CAA TGA CCC CTT CAT TG-TAMRA. Gene expression was analyzed using the ΔΔCT method.

### SDS Gelelectrophoresis and Western Blotting

10 μL plasma depleted whole blood or 2 × 10^7^ cultured CECs were dissolved in Laemmli sample buffer (BioRad, Feldkirchen), sonicated, boiled for 5 min and 25 μg total protein was applied to SDS-PAGE. Western blot analysis was performed using monoclonal anti-HMOX-1 (clone 1F12-A6; Biozol, Eching, Germany), monoclonal anti-FECH (clone D-7; Santa Cruz Biotechnology Inc., Dallas, USA), polyclonal anti-superoxide dismutase (SOD-1, ABIN2913314, antibodies-online, Aachen, Germany), polyclonal anti-glutathion reductase (ABIN5708412, antibodies-online), polyclonal anti-NCOA4 (Biomol GmbH, Hamburg, Germany), monoclonal anti-SARS-CoV-2 nucleocapsid protein (clone 005; Biomol GmbH), monoclonal anti-SARS-CoV-2 spike (clone 42; Biozol, Eching, Germany), monoclonal anti-PPOX (clone 42 J-6; Santa Cruz Biotechnology Inc.), monoclonal anti-UROD (clone C-4; Santa Cruz Biotechnoly Inc.), polyclonal anti-CPOX (Biozol), polyclonal anti-FTH1 (Cell Signaling, Danvers, MA, USA) and monoclonal anti-GAPDH (clone FF26A; eBioscience, as loading control).

### Mass Spectrometry

Erythrocyte porphyrins were extracted from 50 μL plasma-depleted whole blood with 500 μL of acidic methanol solution (1% HCl). Metalloporphyrin was separately extracted with 500 μL acetone pyridine 1 M urea (20:1:1, v/v/v) solution and bilirubin was separately extracted with 500 μL methanol containing 0.1% acetic acid and 0.5% ammonium acetate from another aliquots of blood. Mesoporphyrin IX -dihydrochlorid (Merck) was used as internal standard. The samples were homogenized for 15 min at 4 °C and 300×*g* in a TissueLyser II (Qiagen) after adding 1/3 volume of 0.5 mm metal beads. The resulting mixture was centrifuged at 13,000×*g* for 30 min and 5 μL of the supernatant were added to 200 μL MsMix solution containing: 4:2:1 IP methanol chloroform with 7.5 mM ammoniumformiate.

Mass spectrometric analysis was performed on a Q Exactive spectrometer (Thermo Fischer Scientific, Bremen, DE) equipped with a robotic nanoflow ion source (TriVersa NanoMate, Advion BioSciences, Ithaca, USA) using nanoelectrospray chips with a diameter of 4.1 μm. The ion source was controlled by the Chipsoſt 8.3.1 soſtware (Advion BioSciences). Ionization voltage was +1.5 kV in positive mode; backpressure was set to 1.5 psi. The temperature of the ion transfer capillary was 200 °C; S-lens RF level was set to 50%. All samples were analyzed for 5.7 min. FT MS spectra were acquired within the range of m/z 250–1100 from 0 min to 0.2 min at the mass resolution of R m/z 200 = 140,000; automated gain control (AGC) of 3 × 10^6^ and with the maximal injection time of 3000 ms. t-SIM - 0.2 to 4 min was acquired with R m/z 200 = 140,000; automated gain control of 5 × 10^4^; maximum injection time of 650 ms; isolation window of 20 Th and scan range of m/z 350 to 1100. The inclusion list of masses targeted in t-SIM analyses started at m/z 305 and other masses were computed by adding 10 Th increment starting from m/z 355 up to m/z 905. Parallel reaction monitoring (PRM) spectra were acquired from 4 min to 5.7 min. For PRM, micro scans were set to 1, isolation window to 0.8 Da, normalized collision energy to 12.5%, AGC to 5 × 10^4^ and maximum injection time to 3000 ms. All spectra were pre-processed using repetition rate filtering software PeakStrainer [23] and stitched together by an in-house developed script [24]. Metabolites were identified by LipidXplorer soſtware [25]. Mass accuracy was better than 5 ppm. Metabolites were quantified by comparing the isotopically corrected abundances of their molecular ions with the abundances of internal standards.

### Statistical Analysis

Data were analyzed with GraphPad Prism 6 using Mann-Whitney U test if not otherwise stated. Two-sided P-values of less than 0.05 were considered significant.

### Study Approval

Blood samples from healthy donors and convalescent COVID-19 infected donors were provided by the German Red Cross Donation Service North-East, Institute for Transfusion Medicine (Dresden) and were used in accordance with the guidelines approved by the institutional ethics committee. Blood from COVID-19 infected patients was provided from HELIOS Clinic Aue (Germany). Informed consent was obtained from all donors and patients.

## Results

### Erythroid Progenitor Cells Express the SARS-CoV-2 Entry Receptors ACE2, CD147 and CD26

**Fig. 1:**
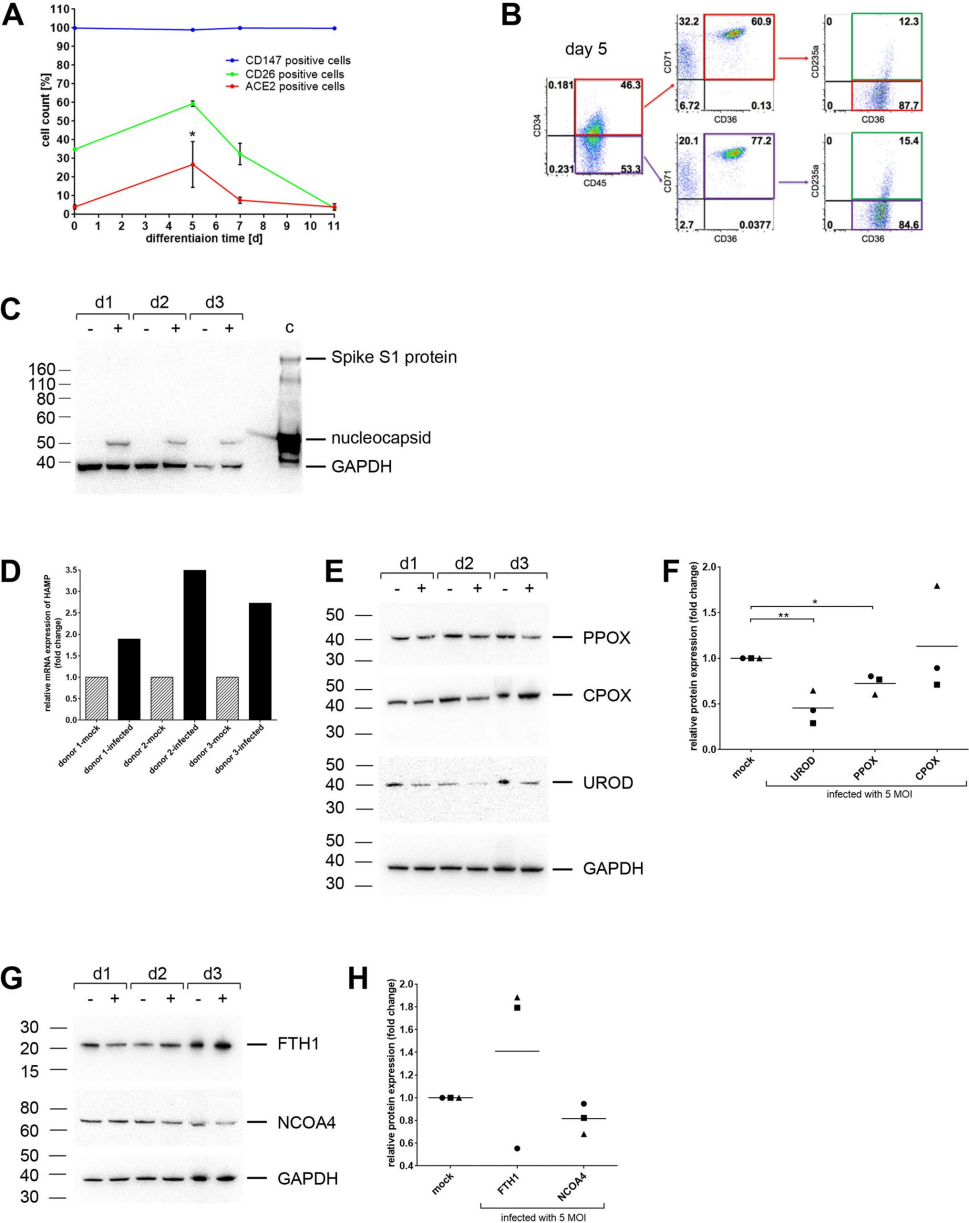
**Infection of CECs with SARS-CoV-2 leads to alterations in hemoglobin synthesis and iron dysmetabolism**. **a** Flow cytometric expression analysis of SARS-CoV-2 entry receptors. CD34^+^ HSCs of three independent donors were differentiated to erythroid cells for 11 days and stained at indicated time points with fluorochrome labeled antibodies against ACE2, CD147, and CD26. **b** Flow cytometric analysis of surface marker expression to determine the cell subpopulation on day 5 of erythroid differentiation. Proerythroblasts are CD34^low/-^CD45^+^CD71^+^CD36^+^CD235a^−^ (red and purple squares) and basophilic erythroblasts are CD34^low/-^CD45^+^CD71^+^CD36^+^CD235a^+^ (green square). **c** Western blot analysis of SARS-CoV-2 infected CECs using anti-nucleocapsid and anti-spike S1 protein antibodies. Inactivated virus particles serve as positive control (c). Polyclonal anti-GAPDH (Cell Signaling, Danvers, MA, USA) was used as loading control. **d** Relative expression of HAMP mRNA to the ΔCT from uninfected cells (mock). Quantitative PCR was performed 2 days after infection with SARS-CoV-2. Relative fold changes in expression (normalized to GAPDH) were calculated by the ΔΔCT method and values are expressed as 2^-ΔΔCT^. Three independent donors are shown. **e** Western blot analysis from CECs derived from CD34^+^ HSPCs (day 7 of differentiation) infected with SARS-CoV-2 or control (mock) using antibodies against UROD, CPOX, PPOX, and GAPDH as loading control. Three independent donors are shown. **f** Quantification of protein expression (UROD, CPOX, PPOX) by western blot analyses (*n* = 3 independent donors). **g** Western blot analysis from CECs derived from CD34^+^ HSPCs (day 7 of differentiation) infected with SARS-CoV-2 or control (mock) using antibodies against FTH1, NCOA4, and GAPDH as loading control. Three independent donors are shown. **h** Quantification of protein expression (FTH1, NCOA4) by western blot analyses (*n* = 3 independent donors). Data are presented as mean ± SEM (standard error mean). Tests were performed two-sided. * p < 0.05, Student *t* test

First, we examined whether CD34^+^ hematopoietic stem cells (HSPCs) and CECs express SARS-CoV-2 entry receptors and are thus potentially susceptible to SARS-CoV-2. Whereas CD147 was expressed in both HSPCs and all types of erythroid cells, ACE2 and CD26 expression was observed in a subpopulation of CECs, with the highest expression at day 5 of in vitro erythroid differentiation, before it dropped back to a low expression level (Fig. 1a). At this time point, the predominant cell types were early erythroid progenitors, which are CD34^+^CD71^+^CD235a^−^, and proerythroblasts, which are CD34^−^CD71^+^CD235a^−^ (Fig. 1b).

### Infection of CECs with SARS-CoV-2 Leads to Impaired Hemoglobin Synthesis and Iron Dysmetabolism

We next performed in vitro erythroid differentiation of CD34^+^ HSPCs of three independent donors and infected the cells on day 5 with MOI 5 of the SARS-CoV-2 B.1.1.7/Alpha variant for 2 days. We observed that CECs are susceptible to SARS-CoV-2 infection as shown by the detection of the nucleocapsid protein (Fig. 1c). Since we found no infectious virus particles in the culture supernatant using plaque assay and a very low expression of spike S1 protein in western blot, it is likely that the virus can infect the cells but is unable to replicate in them. However, there was a strong upregulation of the mRNA levels of hepcidin antimicrobial peptide (HAMP), which codes for hepcidin, the main regulator of iron homeostasis [10], after SARS-CoV-2 infection in the CECs of all donors (Fig. 1d). Furthermore, we assessed the protein expression of key enzymes involved in heme biosynthesis (Fig. 1e, f). Whereas the expression of uroporphyrinogen-decarboxylase (UROD) and protoporphyrinogen-oxidase (PPOX) were downregulated after SARS-CoV-2 infection in CECs of all donors, the expression of coproporphyrinogen-oxidase (CPOX) was downregulated only in CECs of two donors. Cells of donor 3 showed an upregulation of CPOX. Additionally, CECs of donor 2 and 3 showed an upregulation of ferritin heavy chain 1 (FTH1) and a corresponding downregulation of nuclear receptor coactivator 4 (NCOA4), which is involved in the ferritin degradation (ferritinophagy) [26] (Fig. 1g, h). Cells of donor 1 showed no regulation of FTH1 and NCOA4.

### COVID-19 Patients Exhibit Significant Changes in Hematological Parameters and Iron Metabolism

To confirm our in vitro data, we now analyzed the heme and iron metabolism in blood samples from intensive care COVID-19 patients compared to healthy donors and convalescent patients. We first evaluated different hematological parameters and found significant decreases in the hemoglobin content, the amounts of RBCs, the hematocrit, and peripheral lymphocyte counts in the blood samples of COVID-19 patients as compared to healthy donors as well as convalescent COVID-19 patients (Table 1). In contrast, the mean corpuscular hemoglobin (MCH) in the blood of COVID-19 patients was significantly higher compared to both healthy donors and convalescent donors. Since erythrocytes are responsible for the transport of oxygen and carbon dioxide, we further analyzed different blood gas values (pO_2_, pCO_2_, SO_2_, and the parameters of acid-base balance). The pO_2_ from whole blood of severe COVID-19 patients was significant decreased compared to the pO_2_ from healthy donors, whereas the pCO_2_ was unaffected by the SARS-CoV-2 infection. No alterations of the pH were observed, but base excesses and bicarbonate were tendentially increased in COVID-19 patients compared to healthy donors (Table 1). Parameters of iron metabolism also were altered in the plasma of COVID-19 patients compared to healthy donors and convalescent patients. While the inflammatory marker and iron-storage protein ferritin was strongly increased, the plasma iron and transferrin levels were decreased in COVID-19 patients (Table 1).

**Table 1:** Hematological parameters and parameters of iron metabolism in intensive care COVID-19 patients compared to healthy donors and convalescent patients

	COVID patients	Healthy donors	convalescent patients	*p* value heathy vs. COVID	*p* value convalescent vs. COVID
Blood parameters	***n*** **= 27**	***n*** **= 23**	***n*** **= 54**		
hemoglobin [mmol/L]	**6.96 ± 0.24**	**9.04 ± 0.14**	**8.99 ± 0.10**	< 0.0001****	< 0.0001****
red blood cells [10^12^/L]	**3.62 ± 0.13**	**4.87 ± 0.09**	**4.86 ± 0.05**	< 0.0001****	< 0.0001****
hematocrit	**0.33 ± 0.01**	**0.433 ± 0.01**	**0.436 ± 0.004**	< 0.0001****	< 0.0001****
lymphocytes [10^9^/L]	**1.30 ± 0.54**	**2.09 ± 0.11**	**1.94 ± 0.08**	0.035**	0.0027**
thrombocytes [10^9^/L]	255.6 ± 20.78	259.4 ± 8.77	245.4 ± 5.43	0.709	0.329
monocytes [10^9^/L]	0.61 ± 0.06	0.602 ± 0.04	0.550 ± 0.02	0.911	0.637
MCV [fL]	91.69 ± 1.40	89.57 ± 1.00	89.66 ± 0.44	0.322	0.340
MCH [pg]	**31.15 ± 0.51**	**29.54 ± 0.40**	**29.90 ± 0.19**	0.017*	0.021*
Blood gas parameters	***n*** **= 21**	***n*** **= 6**	**n. m.**		
pO_2_ [kPa]	**9.61 ± 0.69**	**14.78 ± 1.53**		0.0004***	
pCO_2_ [kPa]	4.54 ± 0.14	4.47 ± 0.32		0.598	
pH	7.45 ± 0.008	7.42 ± 0.008		0.072	
sO_2_ [%]	**93.71 ± 0.88**	**98.08 ± 0.22**		0.0016**	
BI value [mmol/]	**24.98 ± 0.61**	**22.87 ± 0.67**		0.0418*	
BE value [mmol/]	0.331 ± 0.80	−2.40 ± 1.00		0.0921	
iron metabolism	***n*** **= 27**	***n*** **= 23**	***n*** **= 54**		
plasma ferritin [μg/L]	**1082.7 ± 198.2**	**103.2 ± 20.2**	**131.4 ± 14.4**	<0.0001****	<0.0001****
plasma iron [μmol/L]	**5.21 ± 0.62**	**8.59 ± 0.65**	**9.33 ± 0.61**	0.0004***	<0.0001****
plasma transferrin [g/L]	**1.55 ± 0.07**	**2.60 ± 0.10**	**2.46 ± 0.61**	<0.0001***	<0.0001****

Data are presented as mean ± standard error mean (SEM). Tests were performed two-sided. Mann-Whitney U-test was used for statistical analyses

The bold entries represent significant differences between the sample groups

### Raman Spectra of Erythrocytes from COVID-19 Patients Show Significantly Altered Hemoglobin–Associated Vibrational Modes Compared to Healthy Donors

**Fig. 2:**
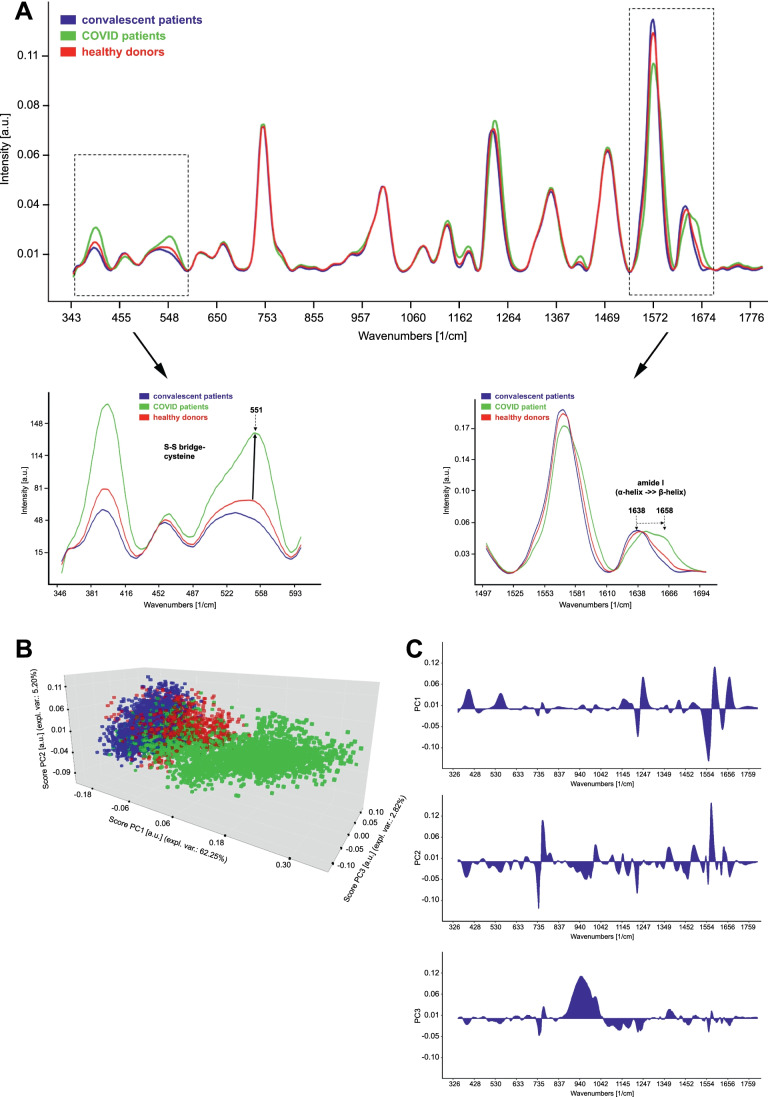
**Raman spectra of erythrocytes from COVID-19 patients reveal significantly altered hemoglobin–associated vibrational modes compared to healthy donors**. **a** Raman mean spectra of erythrocytes from healthy donors, COVID-19 patients, and convalescent patients. Dashed boxes indicate wave number ranges with marked changes of hemoglobin–associated vibrational modes. **b** Principal components analysis (PCA) was conducted on the Raman data. The scores of the PCA can distinctly discriminate the COVID-19 patients (green) from healthy (red) and convalescent patients (blue). **c** Loading blots of PC1 to PC3. These plots show, which parts of the Raman spectra are important for the distribution of the data in the scores plot. The higher a peak, the more important is it

Since hemoglobin is the major component in the erythrocytes, Raman spectra collected from the erythrocytes represent the different vibrational modes of the hemoglobin. As depicted in Fig. 2a, Raman mean spectra of erythrocytes from healthy donors, COVID-19 patients, and convalescent patients show characteristic Raman bands at 1658 (amide I, protein), 1638 (ν(CαCm)asym), 1570 (CβCβ stretching), 1473 (δCH2/CH3), 1354 (symmetric pyrrole half-ring vibration), 1234 (Methine Cm–H deformation), 1180 (asym. Pyrrole half-ring vibration), 1138 (δ(=CbH2)4), 1002 (Phenylalanine), 750 (pyrrole ring def, Tryptophan), 665 (C–S stretch), 551 (S-S stretching), and 396 cm^−1^ (Fe–N stretching vibrations) [27–30]. In COVID-19 patients we found a decrease of the 1638 cm^−1^ and an increase of the 1658 cm^−1^ bands, indicating a change in the spin state and secondary structure of the hemoglobin. Furthermore, an increase in the 396 and 551 cm^−1^ bands was observed. This may indicate the formation of the disulfide bridges in the hemoglobin of the COVID-19 patients [29]. Principal components analysis (PCA) was conducted on the Raman data (Fig. 2b). The scores of the PCA distinctly discriminate the COVID-19 patients (green) from healthy (red) and convalescent patients (blue). The loading plots of the principal components (PC1, PC2, and PC3) reveal the pronounced changes in the Raman spectra between the COVID-19 patients, healthy donors, and convalescent patients. Most of the Raman spectral changes are detected at 1638-1658, 1570, 1234, 551, and 396 cm^-1^ (Fig. 2c).

### COVID-19 Patients Exhibit an Impaired Hemoglobin Metabolism

**Fig. 3:**
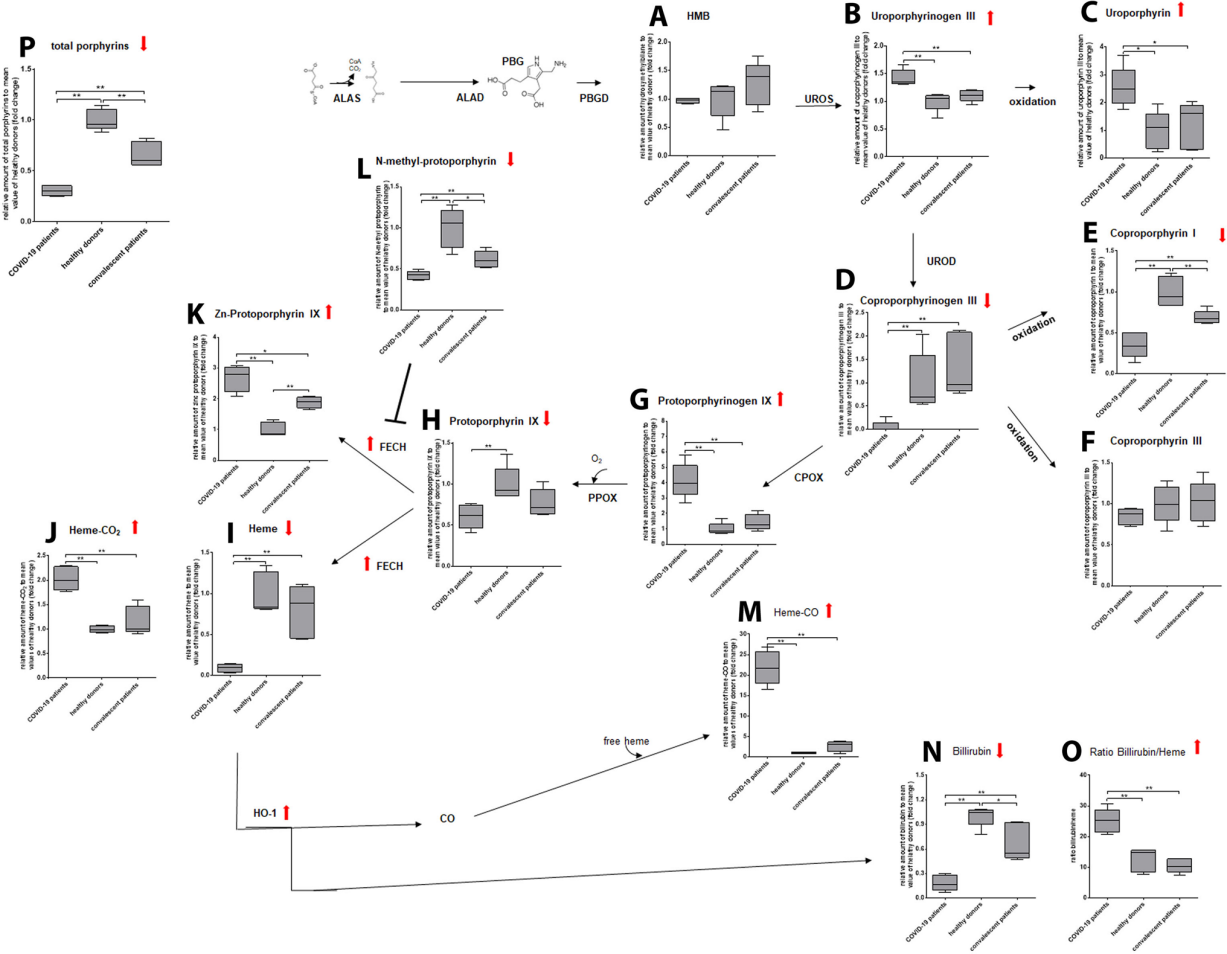
**COVID-19 patients show an altered hemoglobin metabolism.** Mass spectrometric analysis of metabolic intermediates of both heme biosynthesis and degradation in plasma reduced whole blood samples of COVID-19 patients compared to healthy donors and convalescent patients. Relative expressions to the mean value of healthy donors are shown. **a** Hydroxymethylbilane, **b** Uroporphyrinogen III, **c** Uroporphyrin, **d** Coproporphyrinogen III, **e** Coproporphyrin I, **f** Coproporphyrin III, **g** Protoporphyrinogen IX, **h** Protoporphyrin IX, **i** Fe-Heme, **j** heme-CO_2_, **k** Zinc- Protoporphyrin IX, **l** N-Methyl-Protoporphyrin, **m** Heme-CO, **n** Bilirubin, **o** Ratio Bilirubin/Fe-Heme, **p** total porphyrin. *n* = 5 independent donors per group. Boxes with Min and Max are shown. Tests were performed two-sided. * p < 0.05, ** p < 0.01, Mann-Whitney U test

Based on our Raman data, which propose hemoglobin dysfunction, and the increase in ferritin in the COVID-19 patients, we next analyzed the metabolic intermediates involved in hemoglobin synthesis as well as heme degradation using mass spectrometry. While we found no alteration in hydroxymethylbilane levels (Fig. 3a), levels of uroporphyrinogen III and its degradation product uroporphyrin were significantly enhanced in plasma depleted whole blood samples of COVID-19 patients compared to healthy donors and convalescent patients (Fig. 3b, c). In contrast, coproporphyrinogen III, which derives from uroporphyrinogen III, and its degradation product coproporphyrin I, were significantly decreased in blood samples of COVID-19 patients, while coproporphyrin III levels remained unchanged (Fig. 3d-f). Protoporphyrinogen IX, which is synthesized from coproporphyrinogen III, had significantly increased levels in blood samples of COVID-19 patients (Fig. 3g), but the oxidized protoporphyrin IX was detected at significantly lower levels compared to blood from healthy donors and convalescent donors (Fig. 3h). Furthermore, formation of Fe-heme was strongly reduced in blood samples from COVID-19 patients compared to control samples (Fig. 3i), while heme-CO_2_ was increased twofold (Fig. 3j). The formation of heme by transferring iron to protoporphyrin IX is catalyzed by the enzyme ferrochelatase. It was reported that zinc becomes an alternative metal substrate for ferrochelatase in response to iron depletion or impaired iron utilization, leading to increased zinc protoporphyrin IX (ZnPP) formation [31]. Therefore, we also evaluated the ZnPP level in the blood samples. COVID-19 and to a lesser extent convalescent patients displayed strongly increased ZnPP level compared to samples of healthy donors (Fig. 3k). In addition, the inhibitor of ferrochelatase, N-methyl-protoporphyrin, was strongly decreased in COVID-19 patients and less so in convalescents (Fig. 3l). Regarding heme degradation, we were able to detect a strong increase in heme-CO levels in samples of COVID-19 patients compared to control samples (Fig. 3m). Although levels of bilirubin were significantly decreased in samples of COVID-19 patients (Fig. 3n), in relation to Fe-heme they are significantly increased in COVID patients compared to the control groups (Fig. 3o). Of note, the total amount of porphyrin in the samples of COVID-19 patients also was significantly lower than in the control samples (Fig. 3p).

### COVID-19 Patients Show Enhanced Expression of Enzymes Involved in Iron and Hemoglobin Metabolism

**Fig. 4:**
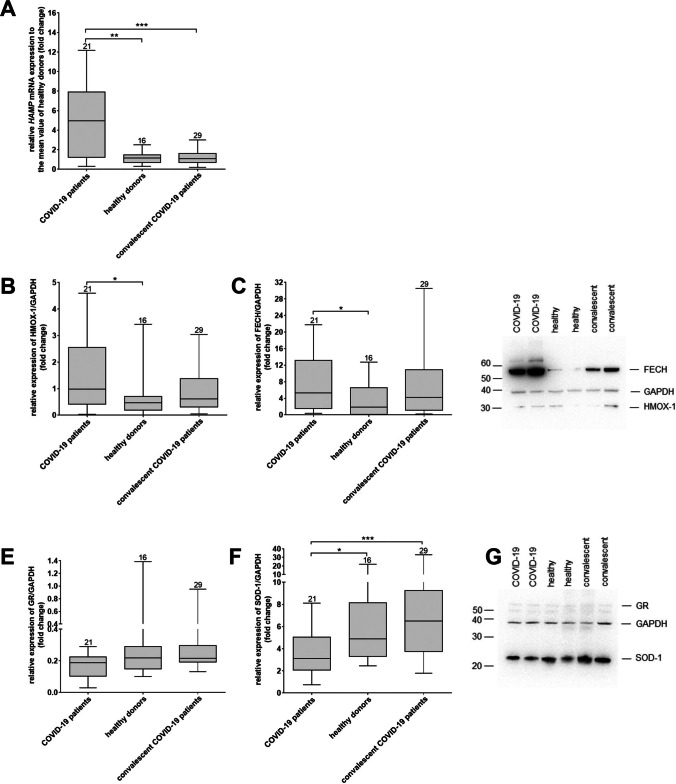
**COVID-19 patients show enhanced expression of enzymes involved in iron and hemoglobin metabolism**. **a** Relative expression of HAMP mRNA to the mean ΔCT value healthy donors. Relative fold changes in expression (normalized to GAPDH) were calculated by the ΔΔCT method and values are expressed as 2^-ΔΔCT^. **b**-**c** Quantification of heme oxygenase (HMOX)-1 (**b**) and ferrochelatase (FECH) (**c**) protein expression by western blot analyses. **d** Western blot analysis of plasma depleted whole blood samples from COVID-19 patients, healthy donors and convalescent patients using antibodies against HMOX-1, FECH, and GAPDH as loading control. Two donors per group are shown. **e**-**f** Quantification of glutathione reductase (GR) (**e**) and superoxide dismutase (SOD)-1 (**f**) protein expression by western blot analyses. **g** Western blot analysis of plasma reduced whole blood samples from COVID-19 patients, healthy donors and convalescent patients using antibodies against GR, SOD-1, and GAPDH as loading control. Two donors per group are shown. **a**, **b**-**c**, **e**-**f** Boxes with Min and Max are shown. The number of analyzed donors are indicated above the boxes.* p < 0.05, *** p < 0.001, Mann-Whitney U test

Due to the decreased iron level in the serum of COVID patients, we next evaluated the expression of key enzymes involved in iron metabolism and hemoglobin biosynthesis at mRNA and protein level. We found a strong increase in HAMP mRNA levels in blood samples of COVID-19 patients compared to healthy donors and convalescent patients (Fig. 4a). Furthermore, we found increased protein levels of hemoxygenase I, which degrades heme to carbon monoxide (CO), iron, and biliverdin as well as ferrochelase, which catalyzes the formation of heme by transferring iron to protoporphyrin-IX [32], in samples of COVID-19 patients compared to healthy donors and convalescent patients (Fig. 4b-d). Regarding redox enzymes, superoxide dismutase-1 (SOD-1), but not glutathione reductase protein expression was significantly decreased in samples from COVID-19 patients compared to healthy donors and convalescent patients (Fig. 4e-g).

## Discussion

Severe COVID-19 is mainly characterized by pneumonia and atypical acute respiratory distress syndrome (ARDS). Complications may include acute liver, cardiac and kidney injury, as well as secondary infection and systemic inflammatory response [33]. Here, we show that hemoglobinopathy and iron overload may play an important, hitherto underrecognized role in COVID-19 pathophysiology. We demonstrate that SARS-CoV-2 can directly infect erythroid precursor cells and that this affects hemoglobin biosynthesis during red blood cell maturation. Moreover, we describe alterations in hemoglobin structure in blood samples of severe COVID-19 patients. Lastly, we show that COVID-19 leads to impaired porphyrin metabolism and changes in the expression of key enzymes involved in iron and hemoglobin metabolism.

The ability of SARS-CoV-2 to infect erythroid progenitor cells is consistent with reports by other research groups [20, 21, 34, 35], as they express the entry receptors for the virus ACE2, CD147 and CD26 [11–13]. However, no viral particles were detected in the cell culture supernatant 2 days after infection, which indicates that the virus cannot replicate in these cells. Nevertheless, infection of erythroid precursor cells with SARS-CoV-2 had direct effects on O2 uptake and transportation due to impaired iron bioavailability and heme biosynthesis. This was shown by a strong increase in HAMP mRNA levels after infection of the cells. HAMP encodes for hepcidin, which is the principal regulator of iron homeostasis [36]. Furthermore, we found the same enhancement of HAMP mRNA levels in the blood of severe COVID-19 patients. High hepcidin levels result in iron sequestration, typically within cytosolic ferritin [36], which we could confirm by an increase of the ferritin heavy chain 1 in infected CECs derived from HSPCs of two independent donors. Additionally, NCOA4, targeting ferritin to autolysomal degradation to release iron [26], was significantly downregulated after infection with SARS-CoV-2. The importance of NCOA4 in intracellular iron homeostasis, hemoglobinization and erythropoiesis was already shown by NCOA4 depletion in the K562 human erythroleukemia cell line, an in vitro model of erythroid differentiation [37] and in a murine cellular model of erythropoiesis, showing defects in hemoglobinization after NCOA4 depletion without affecting differentiation [38]. Besides that, we also found decreased expression of enzymes involved in heme biosynthesis further confirming this hypothesis. Our observations support the hypothesis that the cells activate a mechanism within the innate immune defense to reduce bioavailability of iron to prevent an increasing viral load [10], on the one hand by storing free iron in ferritin and on the other hand by downregulation of the heme biosynthesis. The downregulation of NCOA4 may further provide the explanation for the expansion of CECs in peripheral blood of severe COVID-19 patients, as this protein was identified as a critical regulator of terminal erythroid differentiation [39].

CECs are able to produce reactive oxygen species (ROS) [19], which facilitate hemolysis and release of hemoglobin. Using Raman microscopic analysis, we found changes in spin state of the iron in hemoglobin [40] as well as the tertiary structure shown by the formation of disulfide bridges in samples of COVID-19 patients [29]. Levels of heme-CO_2_ were 2-fold higher in samples of COVID-19 patients compared to control groups. This is in line with the observed increase in bicarbonate levels in COVID-19 patients. Additionally, the levels of heme-CO were increased, indicating an increased degradation of hemoglobin. The results were confirmed by enhanced expression of HMOX-1, which degrades heme to carbon monoxide (CO), iron and biliverdin [41]. The fact, that protoporphyrin IX was decreased in samples from COVID-19 patients could have two reasons. Firstly, we analyzed the cellular fraction of the blood samples only. However, San Juan et al. reported abnormal accumulation of porphyrins in the serum fraction of severe Covid-19 patients [2]. Secondly, we also found an increased expression of FECH, which normally catalyzes the insertion of iron into protoporphyrin IX to form heme [42]. In case of iron deficiency as observed in serum samples of severe COVID-19 patients, FECH was reported to catalyze the incorporation of other metals like zinc instead of iron into protoporphyrin IX, resulting in the formation of zinc protoporphyrin (ZnPP) [43]. This fits well to the observed increase of ZnPP levels in COVID patients compared to healthy donors. The significantly lower level of N-methyl protoporphyrin in COVID-19 patients further supports these results, as it is known as a potent inhibitor of FECH [44]. Regarding redox enzymes, we found no regulation of GR but a downregulation of SOD-1 in samples of COVID-19 patients compared to control groups, suggesting the possible degradation of this enzyme in RBCs from COVID-19 patients, which is in line with other reports [5]. SOD-1 is considered an antioxidant enzyme by catalyzing the conversion of superoxide radicals (O_2_^−^) to hydrogen peroxide (H_2_O_2_) and O_2_ [45].

Taken together, our observations provide further evidence for an important role of direct virus infection of CECs in severe cases of COVID-19 and build upon previous publications showing the susceptibility of hematopoietic and very small embryonic like cells towards SARS-CoV-2 infections [17, 34]. In COVID-19 patients, the number of CECs negatively correlates with the percentage of T-cells and antibody-secreting plasmablasts [21], resulting in suppression of the immune response. Therefore, experimental therapeutic interventions could include anti-oxidative strategies [7] as well as inhibitors of hepcidin [46] to release iron for an effective hemoglobin production. Glucocorticoids are already standard in the treatment of severe COVID-19 [47]. Improved maturation of expanded CECs and downregulation of ACE2 [21] may well contribute to their beneficial effects in severe COVID-19 patients.

## Data Availability

Data and materials are available and can be made available.
